# Ovarian Vein Thrombosis as an Uncommon Cause of Postpartum Fever: A Case Report

**DOI:** 10.7759/cureus.22504

**Published:** 2022-02-22

**Authors:** Junpei Komagamine, Chika Takarada, Taku Yabuki

**Affiliations:** 1 Internal Medicine, National Hospital Organization Tochigi Medical Center, Utsunomiya, JPN

**Keywords:** postpartum women, anticoagulant therapy, diagnostic delay, postpartum fever, ovarian vein thrombosis

## Abstract

Postpartum ovarian vein thrombosis (POVT) is an uncommon cause of postpartum fever. Because POVT is sometimes complicated by pulmonary embolism, preventing diagnostic delay is critical. Nonetheless, the diagnostic delay of POVT is common due to its rarity. Antibiotics along with anticoagulants are recommended as the standard therapy for POVT, but this recommendation is based on older, low-quality literature. Here, we present a case of POVT, presenting with a persistent postpartum fever, which was treated by anticoagulants without antibiotics. Our case highlights the importance of awareness of POVT as a differential diagnosis and the need for studies to investigate the role of antibiotics in POVT.

## Introduction

Postpartum ovarian vein thrombosis (POVT) is an uncommon disease [1]. Although the causes of ovarian vein thrombosis vary, its etiology has been distinguished from other causes of ovarian vein thrombosis, such as cancer [2-4]. The estimated incidence of POVT is reported to be 0.01-0.02% after vaginal delivery and 0.1% after cesarean section [2,5-7]. The classic triad of POVT symptoms is fever, lower abdominal pain, and abdominal mass [2,3]; however, a recent study of POVT reported that abdominal mass was uncommon among patients with POVT [7]. Due to anatomical reasons, most cases of POVT occur in the right ovarian vein [3]. Although POVT can be diagnosed by ultrasound, contrast-enhanced computed tomography (CT) or magnetic resonance imaging (MRI) is more accurate than ultrasound [8-10]. Given that a substantial proportion of POVT patients develop pulmonary embolism [2], which is a common complication, early diagnosis and appropriate treatment are critical for avoiding a poor prognosis for POVT patients who develop pulmonary embolism. Nonetheless, delayed diagnosis of POVT is common due to physicians’ unawareness of the disease [2,3]. Therefore, it is important to consider that POVT is a cause of postpartum fever.

Historically, in 1912, Huggins wrote an article recognizing that Sippel proposed resection of the ovarian vein and uterus in 1894 [11]. Then, Freund was the first to resect the ovarian vein in patients with POVT in 1898, and Trendelenburg performed the first successful resection of the ovarian vein in 1902 [11]. However, since the advent of antibiotics and anticoagulants in the 1940s, POVT treatment has gradually shifted from surgical intervention to medical therapy. A recent Canadian guideline recommends broad-spectrum antibiotics plus anticoagulants as a standard treatment for POVT [12]. However, these recommendations are expert opinions that are not evidence-based [7]. There are no randomized controlled trials (RCTs) investigating the efficacy of antibiotics in POVT [1,10]. Antibiotics have been used for POVT based on the hypothesis that, in most cases, infectious diseases such as endometritis would cause thrombosis in the ovarian vein [1]. However, overt infectious diseases are often undiagnosed in cases of POVT [2,13,14]. Moreover, a substantial proportion of endometritis cases, which are often reported as a cause of POVT, may be misdiagnosed [1,5]. Given that unnecessary use of broad-spectrum antibiotics can result in the emergence of antibiotic-resistant microorganisms, antibiotics need to be reserved for cases of POVT that are truly complicated with an infectious disease.

Here, we report a case of POVT presenting with postpartum fever that was treated with anticoagulants without antibiotics.

## Case presentation

A 35-year-old, otherwise, healthy woman, gravida 3 para 2, was admitted to our hospital because of a persistent fever after vaginal delivery. She had no family history of thrombotic diseases. Twelve days before admission, she had a spontaneous vaginal delivery at full term without any problems and noticed a low-grade fever. Eight days before admission, she was discharged without any interventions from an obstetric clinic. Five days before admission, her temperature suddenly increased to approximately 39.0°C. Because febrile patients could not visit the obstetric clinic without negative results of polymerase chain reaction or antigen test for coronavirus disease 2019, she visited an internal medicine clinic. The primary care physician recommended observation without any diagnosis and prescribed acetaminophen. However, a spiking fever continued, and she presented to our hospital. She reported mild headache, back pain, and mild discomfort during urination but no breast pain, rhinorrhea, sore throat, cough, chest pain, dyspnea, or vaginal discharge. When she was asked about abdominal pain, she reported left lower abdominal pain only during walking.

On presentation, her general appearance was good, and she was alert and oriented. Her temperature was 37.3°C; blood pressure was 108/64 mmHg; pulse was 75 beats per minute; and her oxygen saturation was 100% on ambient air. On examination, there was tenderness in the left lower quadrant of the abdomen with rebound. There was no costovertebral angle tenderness, and the results of other physical examinations were not significant. Gynecological examination revealed no abnormal findings. Therefore, observation and evaluation to determine other nongynecological causes were recommended. Laboratory findings showed an elevated white cell count, C-reactive protein level, and D-dimer level, but other parameters were not significant (Table 1).

**Table 1:** Summary of the laboratory findings.

Variables		Reference range
Blood		
Hematocrit (%)	36.9	35.2–44.4
Hemoglobin (g/dL)	11.5	22.6–14.8
White cell count (per mm^3^)	9,000	3,300–8,600
Platelet count (per mm^3^)	374,000	158,000–348,000
Sodium (mmol/L)	141	138–145
Potassium (mmol/L)	4.1	3.6–4.8
Chloride (mmol/L)	107	101–108
Urea nitrogen (mg/dL)	6.7	8.0–20.0
Creatinine (mg/dL)	0.42	0.46–0.79
Creatine kinase (U/L)	136	41–153
Glucose (mg/dL)	98	73–109
Alanine aminotransferase (U/L)	10	7–23
Aspartame aminotransferase (U/L)	15	13–30
Lactate dehydrogenase (U/L)	227	124–222
C-reactive protein (mg/dL)	14.2	0–0.3
D-dimer (μg/mL)	2.6	<1.0
Antinuclear antibody	Negative at 1:40	Negative
Beta-2 glycoprotein 1 antibodies	Negative	Negative
Anticardiolipin IgG	Negative	Negative
Lupus anticoagulant	Negative	Negative
Protein C (%)	135	64–146
Protein S (%)	69	56–126
Antithrombin III (%)	104	79–121
Urine		
Color	Yellow	Yellow
Clarity	Clear	Clear
Blood	Negative	Negative
Nitrate	Negative	Negative
Leukocyte esterase	Negative	Negative
Leukocytes (per high-power field)	1–4	0–5

**Figure 1:**
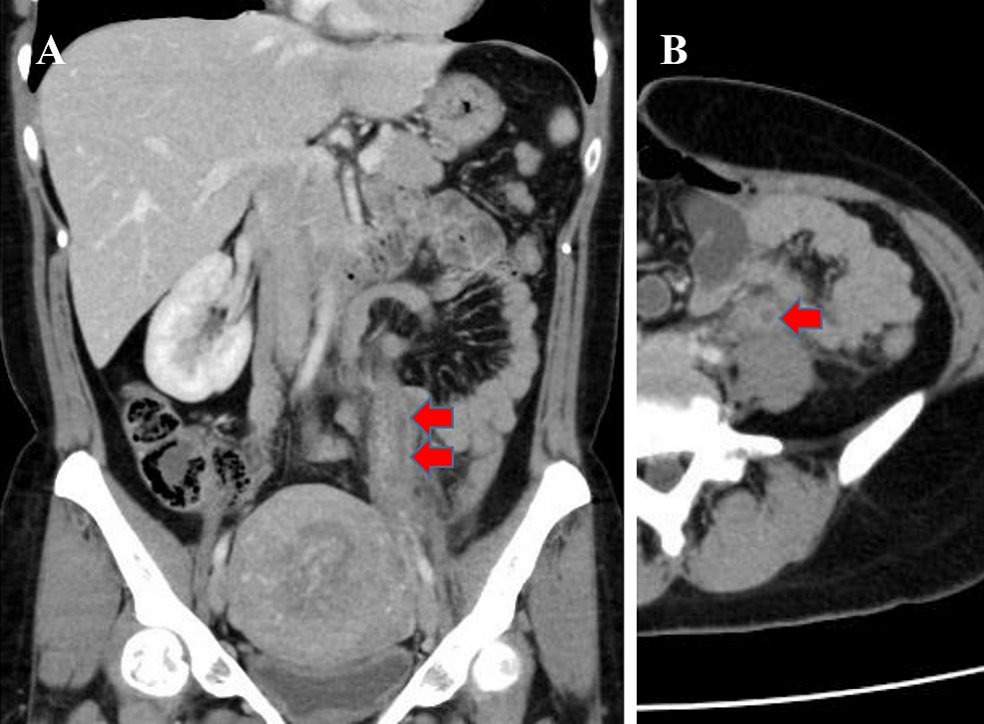
Contrast-enhanced computed tomography of the abdomen showing a thrombus of the left ovarian vein (arrow).

**Figure 2:**
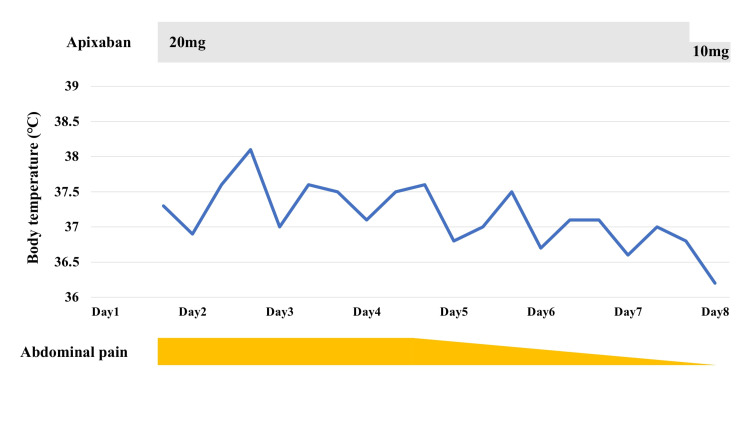
Clinical course.

Urinalysis showed no pyuria. Because peritonitis due to a pelvic infection was suspected, an abdominal CT was performed, which revealed a thrombus of the left ovarian vein (Figure 1). POVT was diagnosed, and apixaban was started. Because blood culture detected no organisms and there were no other symptoms, except fever, that could indicate an infectious disease, antibiotics were not used. Her temperature normalized five days after anticoagulant therapy (Figure 2), and abdominal pain during walking also improved. She was discharged home on the eighth day of her hospital stay. At the follow-up visit a few weeks later, she reported no symptoms. Therefore, apixaban was stopped after treatment for approximately one month. Eight months later, she had no recurrent thrombotic diseases and reported no recurrent symptoms.

## Discussion

This is a case of POVT presenting with persistent postpartum fever. Although POVT was not initially included in the differential diagnosis, fortunately, contrast-enhanced CT ordered for suspected peritonitis due to pelvic infections revealed POVT. Then, because laboratory tests and imaging revealed no apparent source of infection, this patient could be treated with anticoagulants without antibiotics.

Our case teaches us two important lessons. First, POVT should be considered as a differential diagnosis of postpartum fever. Most patients with POVT present with fever and lower abdominal pain [2,3,14], with lower abdominal tenderness often present on examination [2,14]. Moreover, costovertebral angle tenderness has also been reported in case reports [5,15]. In addition, patients with POVT usually do not show abnormal findings during gynecological examination [16]. Therefore, patients with POVT are often misdiagnosed with appendicitis and urinary tract infection [2,3,5,15]. In fact, in our case, although POVT could be diagnosed using contrast-enhanced CT at initial contact, POVT was not included as a differential diagnosis before the imaging tests were performed. Given that there are no specific symptoms or signs of POVT, awareness of this disease as a cause of postpartum fever is critical for preventing delayed diagnosis of POVT.

Second, we were able to treat POVT without antibiotics. In our case, there were no other signs that indicated infectious diseases such as endometritis. Therefore, we did not use antibiotics. Although experts recommend routine broad-spectrum antibiotic therapy as one of the treatments for POVT [12,17], evidence supporting this recommendation is limited [1,7,10]. Moreover, one retrospective study reported that some patients with POVT could be treated without antibiotic therapy [7]. Therefore, antibiotics may not be needed in febrile patients with POVT if they do not have any additional signs that indicate infectious diseases such as endometritis [10]. Nonetheless, in our case, given that the patient showed signs of peritonitis, it was not unreasonable to use empirical antibiotics, first, until POVT was diagnosed.

Anticoagulants, in addition to antibiotics, are recommended as standard therapy for POVT [1,10,12]. Several experts recommend anticoagulant therapy for one to three months [10,12]. However, similar to antibiotics, these recommendations for anticoagulants also lack firm evidence [1,10]. There was only one small RCT that investigated the effect of anticoagulants in patients with POVT [6]. In this trial, there were no differences in the time to become afebrile or the occurrence of thrombotic events between the groups with and without anticoagulants, although the sample size of that study was too small to evaluate the effect of anticoagulants. Moreover, the optimal duration of anticoagulant therapy also remains uncertain. In addition, spontaneous resolution of the thrombus in the ovarian vein without anticoagulant therapy has been reported [6]. Nonetheless, given that thrombotic events recur in a substantial proportion of patients with POVT [1], anticoagulant therapy should be considered in most cases. Further studies are needed to identify and target ideal patients and the duration of therapy regarding anticoagulant therapy.

In most patients with POVT, thrombosis occurs in the right ovarian vein [1], although there is no difference in the occurrence of thrombosis between the right and left ovarian veins in patients with ovarian vein thrombosis due to causes other than pregnancy. However, in our case, we could not explain why thrombosis occurred in the left ovarian vein [18]. There were no anatomical anomalies, no episodes of tendency to take the left lateral decubitus position during pregnancy, and no pelvic mass.

## Conclusions

Ovarian vein thrombosis is an uncommon cause of postpartum fever. Because awareness of POVT is critical for accurate diagnosis, clinicians should consider it as a differential diagnosis for postpartum fever. The standard treatment for POVT is antibiotics along with anticoagulants. However, these recommendations are not supported by high-quality evidence. Whether antibiotics are needed for febrile patients with POVT if they have no additional signs associated with infections remains uncertain. Further studies are needed to identify the optimal use of antibiotics and anticoagulants in patients with POVT.

## References

[1] Dougan C, Phillips R, Harley I, Benson G, Anbazhagan A (2016). Postpartum ovarian vein thrombosis. Obstet Gynaecol.

[2] Dunnihoo DR, Gallaspy JW, Wise RB, Otterson WN (1991). Postpartum ovarian vein thrombophlebitis: a review. Obstet Gynecol Surv.

[3] Munsick RA, Gillanders LA (1981). A review of the syndrome of puerperal ovarian vein thrombophlebitis. Obstet Gynecol Surv.

[4] Virmani V, Kaza R, Sadaf A, Fasih N, Fraser-Hill M (2012). Ultrasound, computed tomography, and magnetic resonance imaging of ovarian vein thrombosis in obstetrical and nonobstetrical patients. Can Assoc Radiol J.

[5] Witlin AG, Sibai BM (1995). Postpartum ovarian vein thrombosis after vaginal delivery: a report of 11 cases. Obstet Gynecol.

[6] Brown CE, Stettler RW, Twickler D, Cunningham FG (1999). Puerperal septic pelvic thrombophlebitis: incidence and response to heparin therapy. Am J Obstet Gynecol.

[7] Rottenstreich A, Da'as N, Kleinstern G, Spectre G, Amsalem H, Kalish Y (2016). Pregnancy and non-pregnancy related ovarian vein thrombosis: clinical course and outcome. Thromb Res.

[8] Twickler DM, Setiawan AT, Evans RS, Erdman WA, Stettler RW, Brown CE, Cunningham FG (1997). Imaging of puerperal septic thrombophlebitis: prospective comparison of MR imaging, CT, and sonography. AJR Am J Roentgenol.

[9] Kubik-Huch RA, Hebisch G, Huch R, Hilfiker P, Debatin JF, Krestin GP (1999). Role of duplex color Doppler ultrasound, computed tomography, and MR angiography in the diagnosis of septic puerperal ovarian vein thrombosis. Abdom Imaging.

[10] Bannow BT, Skeith L (2017). Diagnosis and management of postpartum ovarian vein thrombosis. Hematology Am Soc Hematol Educ Program.

[11] Huggins RR (1912). The ligation or excision of the ovarian or deep pelvic veins in the treatment of puerperal thrombophlebitis. JAMA.

[12] Chan WS, Rey E, Kent NE (2014). Venous thromboembolism and antithrombotic therapy in pregnancy. J Obstet Gynaecol Can.

[13] Salomon O, Dulitzky M, Apter S (2010). New observations in postpartum ovarian vein thrombosis: experience of single center. Blood Coagul Fibrinolysis.

[14] Salomon O, Apter S, Shaham D (1999). Risk factors associated with postpartum ovarian vein thrombosis. Thromb Haemost.

[15] Azhar E, Nguyen T, Waheed A (2020). Left ovarian vein thrombosis presenting as acute postpartum pyelonephritis. Cureus.

[16] Brown TK, Munsick RA (1971). Puerperal ovarian vein thrombophlebitis: a syndrome. Am J Obstet Gynecol.

[17] Abbattista M, Capecchi M, Martinelli I (2020). Treatment of unusual thrombotic manifestations. Blood.

[18] Lenz CJ, Wysokinski WE, Henkin S (2017). Ovarian vein thrombosis: incidence of recurrent venous thromboembolism and survival. Obstet Gynecol.

